# Management of HER2-Positive Early Breast Cancer in Italy: A Maze Presenting Opportunities and Challenges

**DOI:** 10.3389/fonc.2022.871160

**Published:** 2022-05-19

**Authors:** Luigia Stefania Stucci, Marco Pisino, Claudia D’Addario, Teresa Grassi, Angela Toss

**Affiliations:** ^1^ Department of Biomedical Sciences and Human Oncology, Azienda Ospedaliero-Universitaria (A.O.U.), Consorziale Policlinico di Bari, Bari, Italy; ^2^ Department of Biomedical Sciences and Human Oncology, University of Bari Aldo Moro, Bari, Italy; ^3^ Department of Oncology and Haematology, Genetic Oncology Unit, University Hospital of Modena, Modena, Italy

**Keywords:** HER2+ breast cancer, early stage, treatment, Italy management, clinical practice

## Abstract

The management of human epidermal growth factor receptor 2 (HER2)-positive early-stage breast cancer (BC) has changed in recent years thanks to the introduction of anti-HER2 agents in clinical practice as standard of care in the neoadjuvant setting. In this scenario, we probed the issue of which HER2-positive BC patients are eligible for neoadjuvant or for adjuvant treatment, since these therapeutic strategies seem to be mutually exclusive in clinical practice according to an Italian drug surveillance system. We reviewed both alternatives to establish which is more suitable, considering the anti-HER2 drugs available in Italy. Randomized clinical trials demonstrated a similar clinical benefit for chemotherapy administered as neoadjuvant therapy or adjuvant therapy. A meta-analysis, including 11,955 patients treated with neoadjuvant therapy, demonstrated an improvement in event-free survival (EFS) and overall survival (OS). Moreover, the recent APHINITY trial, analyzed at 6 years follow-up, demonstrated the superiority of the combination pertuzumab–trastuzumab versus trastuzumab–placebo in previously untreated patients. A greater benefit was found in patients with positive lymph nodes treated in the adjuvant setting. Our analysis underlines the need for a therapeutic decision-making algorithm, which is still unavailable, to support clinicians in identifying patients suitable for neoadjuvant or adjuvant therapy. Further prospective clinical trials should be performed in collaboration with other Italian Breast Cancer Centers to establish the best strategy to be adopted in early HER2+ BC.

## Introduction

HER2 overexpression is present in 15%–20% of breast cancers (BCs), inducing an aggressive phenotype and poor patient outcomes (1). The introduction of anti-HER2 drugs has dramatically changed the prognosis for these patients. The addition to chemotherapy of trastuzumab, a humanized monoclonal antibody that binds to the extracellular domain of HER2 receptors, significantly improved survival in patients with early-stage HER2-positive BC (2), but despite the improvement in both disease-free survival (DFS) and overall survival (OS) in early HER2-positive BC, long-term follow-up data indicate that approximately 25% of patients still develop disease recurrence (3). Further therapeutic strategies have therefore been explored (4), using other anti-HER2 drugs such as pertuzumab, a monoclonal antibody that blocks another extracellular subdomain of the HER2 receptor, the antibody–drug conjugate trastuzumab–emtansine (T-DM1), and the irreversible pan-HER2 inhibitor neratinib in the neoadjuvant or adjuvant setting for the management of early HER2-positive BC (5).

In Italy, trastuzumab is approved in neoadjuvant treatment for tumors >1 cm or node-positive BC patients and, in cases of a complete pathologic response, as adjuvant therapy. T-DM1 is approved and reimbursed for residual disease after neoadjuvant treatment with trastuzumab, whereas the double blockade with pertuzumab and trastuzumab is approved and reimbursed in the adjuvant setting in Italy only for high-risk HER2+ BC patients (6, 7).

For this reason, two different strategies could be reserved to high-risk early HER2+ BC patients, namely, both neoadjuvant or pre-surgery treatment and adjuvant or post-surgery treatment. These options have different goals, but both are aimed at improving the long-term clinical outcome in these patients.

Neoadjuvant HER2-based therapy is typically used in locally advanced BC (Stage IIb with T3 disease or Stage III) or in patients with an earlier stage HER2+ disease who desire breast-conserving therapy, those who have limited axillary nodal involvement (N1) (which may convert to node-negative disease and thus resulting in sentinel lymph node biopsy), or those whose surgery has been postponed (due to a variety of reasons). Adjuvant chemotherapy is given to patients with HER2+ disease, i.e., node-positive or node-negative disease with tumors >1 cm in size.

The goals of neoadjuvant therapy are mainly to assess *in vivo* complete tumor response [pathologic complete response (pCR)], the potential for less radical surgical treatment, and to evaluate the opportunity of delivering targeted therapies in the residual disease setting. We know that pCR is associated with BC prognosis, and the presence of residual disease after the neoadjuvant therapeutic approach suggests biological resistance, identifying a subgroup population at risk of relapse.

The goals of adjuvant treatment are to improve OS, frequently assessed as 5- and 10-year survival, and to prolong the disease-free interval in patients with early BC.

According to Italian drug regulatory bodies, particularly in node-positive HER2+ BC, we can adopt both neoadjuvant and adjuvant therapy strategies. Therefore, trastuzumab could be used before surgery and trastuzumab or TDM1 in the adjuvant setting in cases of both a complete response and a residual tumor, as well as double blockade of HER2 receptors with pertuzumab and trastuzumab directly in the post-surgery setting in high-risk (node positive) HER2+ untreated BC patients.

However, no criteria are yet available to support the selection of which patients to treat with neoadjuvant or adjuvant treatment, particularly in high-risk (or node-positive) HER2+ BC patients. Moreover, despite the current improved therapeutic options, the emergence of treatment resistance remains a problem, especially in the advanced-stage disease setting. Thus, planning the optimal strategy at an earlier stage could be essential to define the ideal sequence of systemic therapies to be applied in local and advanced disease.

### Neoadjuvant Therapy of HER2+ BC: State of the Art

Neoadjuvant treatment was long reserved for patients with inoperable, locally advanced or inflammatory BC, with the aim of making the tumor operable and also of improving the surgical rates and quality. Furthermore, one of the key objectives of neoadjuvant chemotherapy was the clinical and pathological downstaging of positive axillary lymph nodes. Randomized clinical trials performed in the 1980s and 1990s demonstrated the same survival benefit for chemotherapy administered as neoadjuvant therapy or as adjuvant therapy. Since then, the approach to neoadjuvant treatment has changed and treatment decisions are currently based on the tumor biology and tumor stage. In the context of operable disease, most patients with HER2-positive tumors measuring >1 cm and/or node-positive disease undergo neoadjuvant treatment (4, 8–11). In Italy, the main indications for neoadjuvant chemotherapy currently include the following: locally advanced BC (stages IIB–IIIC), since in most cases these are not susceptible to conservative surgery and because of the higher risk of relapse; early-stage BC (stages I–IIA) if conservative surgery is not feasible, for example, due to a high tumor–breast ratio or if the expected cosmetic outcome is suboptimal due to the particular tumor location.

The main objective of neoadjuvant therapy in BC is to achieve complete response (pCR). By pCR, we mean the absence of residual invasive disease in the breast and the absence of measurable disease in any axillary lymph node (ypT0 ypN0). Since the possible persistence of carcinoma *in situ* does not affect the risk of relapse, the exclusive presence of residual intraductal tumor cells still corresponds to the definition of pCR (ypT0/Tis ypN0). The pCR rate at the time of surgery is associated with a more favorable prognosis and provides information on the reactivity of the tumor to systemic therapy. A pooled analysis defined pCR as the strongest discriminator of long-term outcomes for patients in the neoadjuvant setting (12). In 2014, a meta-analysis including 11,955 patients from 12 randomized clinical trials of neoadjuvant therapy was conducted; at 3-year follow-up (CTNeoBC), eradication of breast and lymph node cancer was associated with an improved EFS (HR 0.48; 95% CI 0.43–0.54) and OS (HR 0.36; 95% CI 0.31–0.42). The association between pCR and long-term outcome was observed in all subtypes of BC (TBNC, HR+/HER2+, and HR-/HER2+). In particular, the pCR rate was higher in rapidly growing tumors, including triple-negative BC (EFS: HR 0.24, 95% CI 0.18–0.33; OS: HR 0.16, 95% CI 0.11–0.25) and HER2-positive BC (EFS: HR 0.39, 95% CI 0.31–0.50; OS: HR 0.34, 95% CI 0.24–0.47). The response to neoadjuvant treatment in HER2-positive BC depends on the state of the hormone receptors. A pCR rate of 30.9% was observed in patients with HER2-positive hormone receptor-positive BC treated with an anti-HER2 agent, as compared with 18.3% treated with chemotherapy alone (HR 0.58; 95% CI 0.42–0.829), and the pCR rate was 50.3% in patients with hormone receptor negative HER2-positive BC treated with an anti-HER2 agent versus 30.2% without a neoadjuvant anti-HER2 agent (HR 0.25; 95% CI 0.18–0.34) (13). The main drugs with an anti-HER2 action include trastuzumab, a humanized monoclonal antibody targeted against the extracellular portion of HER2; lapatinib, an orally bioavailable double tyrosine kinase inhibitor (TKI) specific for HER2 and EGFR/HER1; pertuzumab, a humanized monoclonal antibody that binds to the extracellular dimerization subdomain II of HER2; ado-trastuzumab emtasine (T-DM1), an antibody–drug conjugate of trastuzumab and the cytotoxic agent emtansine (DM1); and neratinib, an oral, irreversible inhibitor of the human epidermal growth factor receptors HER1 (EGFR), HER2, and HER4. In patients with operable HER2-positive tumors, a randomized phase II trial evaluated the addition of trastuzumab to paclitaxel chemotherapy for 4 cycles and FE(75)C for 4 cycles. The study was prematurely closed after enrolling only 42 randomized patients due to the finding of a significant increase in the pCR rate with trastuzumab (65% vs. 26%) (14). Another randomized phase III trial (NOAH trial) compared neoadjuvant chemotherapy plus trastuzumab, followed by 1 year of trastuzumab, with neoadjuvant chemotherapy alone in patients with locally advanced or inflammatory HER2-positive BC. This study demonstrated a statistically significant improvement in total pCR (38 vs. 19%; *p* = 0.001), a 5-year event-free survival (EFS) of 58 vs. 43% (HR 0.64; 95% CI 0.44–0.93; *p* = 0.016) and a non-significant improvement in OS (74 vs. 63%; HR 0.66; *p* = 0.055). Trastuzumab also resulted in a 40% reduction in the risk of relapse, progression, or death compared to chemotherapy alone (15). Further updated data at a median follow-up of 5 years (of trastuzumab-containing neoadjuvant therapy followed by adjuvant trastuzumab) in patients with locally advanced or inflammatory BC highlight the association between a complete response and long-term outcomes of patients with HER2+ disease.

In the neoadjuvant setting, other studies have considered combinations of trastuzumab with lapatinib or pertuzumab. The international randomized Phase III NeoALLTO trial compared lapatinib alone, trastuzumab, or the combination of both, together with paclitaxel (16). Results showed that the combination of trastuzumab and lapatinib plus paclitaxel achieved a pCR of 51.3% versus 29.5% in the trastuzumab plus paclitaxel group and 24.7% in the lapatinib plus paclitaxel group (*p* = 0.0001). However, lapatinib caused worse side effects, mainly rash and diarrhea. Furthermore, the NSABP B-41 study did not show any significant differences between the combination of trastuzumab and lapatinib compared with monotherapy (17). In conclusion, lapatinib as a single agent or in combination with trastuzumab appears to be ineffective and more toxic. NEOSPHERE, a multicenter, international, randomized, controlled phase II study on pertuzumab, was conducted in 417 adult female patients with newly diagnosed, early-stage, inflammatory, or locally advanced HER2-positive (T2-4d; primary tumors >2 cm in diameter), not previously treated with trastuzumab, chemotherapy, or radiotherapy. The primary endpoint was pCR, and the results were as follows: 45.8% pCR for trastuzumab, pertuzumab, and docetaxel; 29.0% pCR for trastuzumab and docetaxel; 24.0% pCR for pertuzumab and docetaxel; and 16.8% pCR for trastuzumab and pertuzumab. The different pCR rate obtained in the patient group treated with pertuzumab + trastuzumab and docetaxel as compared to those treated with trastuzumab and docetaxel likely results in a clinically significant difference in long-term outcomes and is supported by the positive PFS trend (HR 0.69, 95% CI 0.34–1.40) and DFS (HR 0.60, 95% CI 0.28–1.27). The pCR rates and the extent of benefit obtained with pertuzumab were lower in the subgroup of patients with hormone receptor-positive BC (6% difference in pCR) compared to patients with hormone receptor-negative tumors (26.4% difference in pCR) (18). Cardiotoxicity is the most important adverse effect deriving from treatment with anti-HER2 agents and is worse when combined with anthracyclines. Importantly, the addition of pertuzumab did not produce any significant reduction in cardiac function. To foster the use of less cytotoxic regimens in selected patients (stages II–III), some studies have evaluated treatment with pertuzumab and trastuzumab alone (chemotherapy-free regimen). The WGS-ADAPT study observed that neoadjuvant therapy with TDM-1 is more effective in HER2-positive and HR-positive BC, whether or not associated with endocrine therapy, than treatment with trastuzumab plus endocrine therapy. However, the KRISTINE trial showed that TDM-1 + pertuzumab did not perform better than conventional chemotherapy (TCHP) (19, 20). In the phase II NA-PHER2 study, patients with HR-positive and HER2-positive BC were treated every 3 weeks with intravenous trastuzumab and pertuzumab for 6 cycles, plus oral palbociclib and intramuscular fulvestrant every 4 weeks for 5 cycles. Co-primary endpoints were a change from baseline in Ki67 expression at 2 weeks of treatment and surgery (16 weeks post-treatment) and changes in apoptosis from baseline to surgery. Secondary endpoints were the clinical objective response according to RECIST response assessment criteria in solid tumors, and complete response. The combination of palbociclib, fulvestrant, trastuzumab, and pertuzumab had a significant effect on Ki67 expression at 2 weeks and on surgery. At surgery, 8 patients (27%) had a complete response in the mammary and axillary lymph nodes (21). PALTAN [NCT02907918] is an ongoing phase II neoadjuvant trial of palbociclib in combination with letrozole (plus goserelin if premenopausal) and trastuzumab in women with stage II–III ER+ HER2+ BC; results are expected shortly.

### Update on Adjuvant Therapy of HER2+ BC

In HER2-positive BC, trastuzumab has been the standard of care in the adjuvant setting, administered with taxane and maintained for 1 year. The HERA trial (22) established the role of trastuzumab, demonstrating that 1 year of trastuzumab sequentially introduced after adjuvant chemotherapy significantly improved progression-free survival (PFS) (HR 0.54; 95% CI, 0.43–0.67, *p* < 0.0001). Another study, conducted by Ramond et al., compared the use of trastuzumab with adjuvant chemotherapy against chemotherapy alone, showing higher 3-year DFS rates (87.1% versus 75.4%; HR 0.48; 95% CI, 0.39–0.59, *p* < 0.0001) and higher OS, despite an increased incidence of class III and IV congestive heart failure (23). Trastuzumab yielded great benefit among HER2-positive patients. Various de-escalation strategies were tested through the years, reducing either the trastuzumab duration or dosage. Many studies have been carried out to demonstrate the non-inferiority of de-escalation strategies (24–29), evaluating the use of trastuzumab for a shorter period of time. Only the PHERSEPHONE trial demonstrated a non-inferiority of 6 months of trastuzumab compared to 12 months of trastuzumab (HR 1.07; 95% CI, 0.93–1.24, *p* = 0.011). However, 53% of patients in this study did not receive trastuzumab concurrently with chemotherapy, and those who did receive concurrent trastuzumab showed a greater benefit from 1 year of trastuzumab (HR 1.53; 95% CI, 1.16–2.01, *p* = 0.001) (30). In recent years, a new anti-HER2 agent, pertuzumab, has been evaluated in many studies. Pertuzumab has proven to be a valuable therapeutic option in early-stage HER2-positive BC. The use of pertuzumab has been studied in combination with trastuzumab in both adjuvant and neoadjuvant settings with favorable results, demonstrating the superiority of a double anti-HER2 strategy as compared to trastuzumab as a single-agent anti-HER2 therapy.

The APHINITY trial (31) demonstrated the superiority of the combination pertuzumab–trastuzumab versus trastuzumab–placebo in the adjuvant setting, showing higher rates of 3-year invasive disease-free survival (IDFS) in the arm receiving the double anti-HER2 therapy (HR 0.81; 95% CI, 0.66–1.00; *p* = 0.045). A greater benefit was highlighted particularly in the subset of patients with positive lymph nodes (3-year DFS 92% versus 90.2%; HR 1.13; 95% CI, 0.68–1.86; *p* = 0.02). No significant difference in mortality rates was found at the first interim analysis (HR 0.89; 95% CI, 0.66–1.21, *p* = 0.47), as well as no significant increase in cardiotoxicity rates. This trial presented pertuzumab as a valuable and effective additional drug, not burdened by a significant increase in side effects. Based on these results, pertuzumab was approved by the EMA in 2018 in association with chemotherapy and trastuzumab in the adjuvant setting for high-risk HER2-positive BC. More recently, pertuzumab was approved in Italy for patients with high-risk HER2-positive early BC in association with trastuzumab and chemotherapy.

The phase III KATHERINE trial (32) was designed with the objective of identifying the optimal therapeutic strategy in HER2-positive BC among patients who did not achieve pCR after neoadjuvant therapy. The primary endpoint was IDFS, and patients were randomized to receive either T-DM1 or trastuzumab in the adjuvant setting. The arm treated with T-DM1 showed a lower incidence of recurrent invasive disease (12.2% versus 22.2%) and a higher OS (HR 0.5; 95% CI, 0.39–0.64, *p* < 0.001). Consistent benefit was observed among all subgroups of patients receiving T-DM1, independently of previous neoadjuvant anti-HER2 targeted therapy. In the subset treated with neoadjuvant chemotherapy and trastuzumab, invasive disease events occurred in 78 patients in the T-DM1 arm versus 141 patients receiving adjuvant trastuzumab (HR 0.49; 95% CI, 0.37–0.65). Among patients treated with double anti-HER2 agents, invasive disease events occurred in 13 patients in the T-DM1 group versus 24 patients in the trastuzumab arm (HR 0.54; 95% CI, 0.27–1.06). Of the trial participants, 19.5% had been previously treated with double anti-HER2 neoadjuvant therapy, and among these, the combination of pertuzumab and trastuzumab was the preferred strategy (93.8%). As regards safety, 12.7% of patients in the T-DM1 arm suffered serious adverse effects versus 8.1% in the trastuzumab arm, while adverse events of any grade were more common in the T-DM1 arm (98.8% versus 93.3%). Although this trial seems to show a greater efficacy of T-DM1 versus trastuzumab, no comparison has been made between T-DM1 and trastuzumab–pertuzumab in the adjuvant setting.

In Italian practice, T-DM1 has been approved in patients who did not obtain pCR after neoadjuvant trastuzumab. Other adjuvant strategies have been studied in HER2-positive BC, particularly TKIs (neratinib and lapatinib). In the ExtraNet trial (33), 1 year of neratinib was administered after 1 year of adjuvant trastuzumab, improving DFS in ER+/HER2+ patients, while no benefit was observed in ER− patients. In ER+ patients, neratinib was combined with endocrine therapy. The use of lapatinib, another TKI, was investigated in the ALTTO trial (34), alone and in combination with trastuzumab, but did not show any significant improvement of 5-year DFS or OS.

## Discussion

Several studies have demonstrated the efficacy of trastuzumab in improving EFS, pCR, and OS, resulting in a strong correlation between complete response and long-term outcomes of patients with HER2+ disease (15, 35). Despite these encouraging results, 15% of patients still relapse after therapy with trastuzumab due to resistance to trastuzumab (36, 37). Pertuzumab is a drug that has been investigated in patients with HER2+ BC to overcome the resistance. It is a monoclonal antibody that binds to subdomain II of the HER2 receptor and thereby blocks heterodimerization with HER3, subsequently inhibiting downstream signaling. The addition of pertuzumab to trastuzumab in the neoadjuvant setting was assessed in the NEOSPHERE Trial, demonstrating that pertuzumab enhances locoregional responses in patients with locally advanced, inflammatory, or early-stage HER2+ invasive BC (size >2 cm or node-positive disease) (18). Nevertheless, these results, the small sample size, the lack of a blinded pathology review, and the chosen chemotherapy backbone limited the use of pertuzumab in Italy, which is approved but not reimbursed in the neoadjuvant setting. Indeed, based on published results, the APHINITY trial was performed to explore the role of anti-HER2 combination treatment with pertuzumab and trastuzumab in the adjuvant setting. In the Phase III APHINITY trial (31), the addition of pertuzumab to herceptin and chemotherapy led to an improved 3-year DFS (94.1 versus 93.2%; HR 0.81, 95% CI 0.66–1.00); subgroup analysis showed a greater improvement in patients with node-positive disease (92 vs. 90.2%; HR 0.77, 95% CI 0.62–0.96) but no difference in those with node-negative disease. Dual anti-HER2-directed therapy is recommended, approved, and reimbursed for high-risk disease (node-positive or node-negative, tumor size >2 cm) in the adjuvant setting.

Therefore, combination therapy with pertuzumab and trastuzumab in untreated high-risk HER2-positive BC in an early setting may be preferred for various reasons:

1) The effect of neoadjuvant therapy in node-positive HER2+ BC: one of the clinical response criteria in the NOAH trial was complete response (in breast tissue and axillary nodes), obtained in 38% of patients treated with trastuzumab versus placebo; this concept was reinforced in the Neosphere trial, which provided new insights into the association between total pCR and long-term outcomes. The 5-year PFS rates were 85% for patients who achieved pCR compared with 76% in patients who did not achieve pCR (HR 0.54). Thus, the response to neoadjuvant therapy with anti-HER2 agents, evaluating breast tissue and nodes, is more predictive of clinical benefit than evaluating response only on primitive breast tumors. Thus, nevertheless, pertuzumab is more effective in inducing tpCR than trastuzumab in monotherapy; in Italy, this option is not available because it is not reimbursable.
2) Potential loss of HER2 expression after neoadjuvant therapy (NACT): Mittendorf et al. reported a 32% loss of HER2 amplification after NACT plus trastuzumab and Guarneri et al. reported a loss of HER2 expression in 40% of patients after NACT alone and in 14.7% of patients after NACT plus trastuzumab. In both studies, patients with HER2+ tumors at diagnosis but no HER2 amplification/overexpression in residual disease had a worse DFS compared to those who maintained HER2+ residual disease (38, 39).
3) The risk of underestimation of clinical tumor staging after NACT: For clinically axillary lymph node (ALN)-negative patients, the HER2-positive subtype is found to have a high node-negative rate at pathology, and sentinel lymph node biopsy (SLNB) is recommended after NACT. For patients with positive ALNs that convert to negative, the false-negative rate is high. Moreover, in patients with positive ALNs that convert to node-negative after NACT, whether SLNB can replace ALN dissection or not remains controversial (40). In this scenario, there is a risk of underestimating clinical and pathological staging of initial node-positive HER2+ BC. Even though APHINITY trial showed positive results, it is important to outline that the addition of pertuzumab portends only a small benefit in the overall population and a more significant benefit in high-risk, lymph node-positive patients, and should therefore be advised in this group of patients only. For now, there are still no clear guidelines for the use of adjuvant pertuzumab after neoadjuvant use, especially in patients who achieved pCR. The majority of neoadjuvant pertuzumab trials consisted of the single-agent trastuzumab in the adjuvant setting (31).

In node-negative HER2+ BC patients, we know that the addition of trastuzumab to adjuvant chemotherapy reduces the risk of recurrence by approximately 40% and the risk of death by up to 30% (41). According to meta-analyses, the benefits of trastuzumab are independent of age, T and N stage, and hormone receptor status. Importantly, real-world data confirmed a similar benefit (42–44). Despite the significant impact on outcomes of trastuzumab introduction in the adjuvant setting, after a follow-up of 8–11 years, 15%–24% of patients experienced disease recurrence. Moreover, as demonstrated by the randomized phase III KATHERINE trial (32), HER2-positive patients with residual disease after neoadjuvant taxane-containing chemotherapy and trastuzumab could benefit from receiving 14 cycles of T-DM1, leading to an improvement of IDFS (88 vs. 77%; HR 0.50; 95% CI 0.39–0.64).

**Figure 1:**
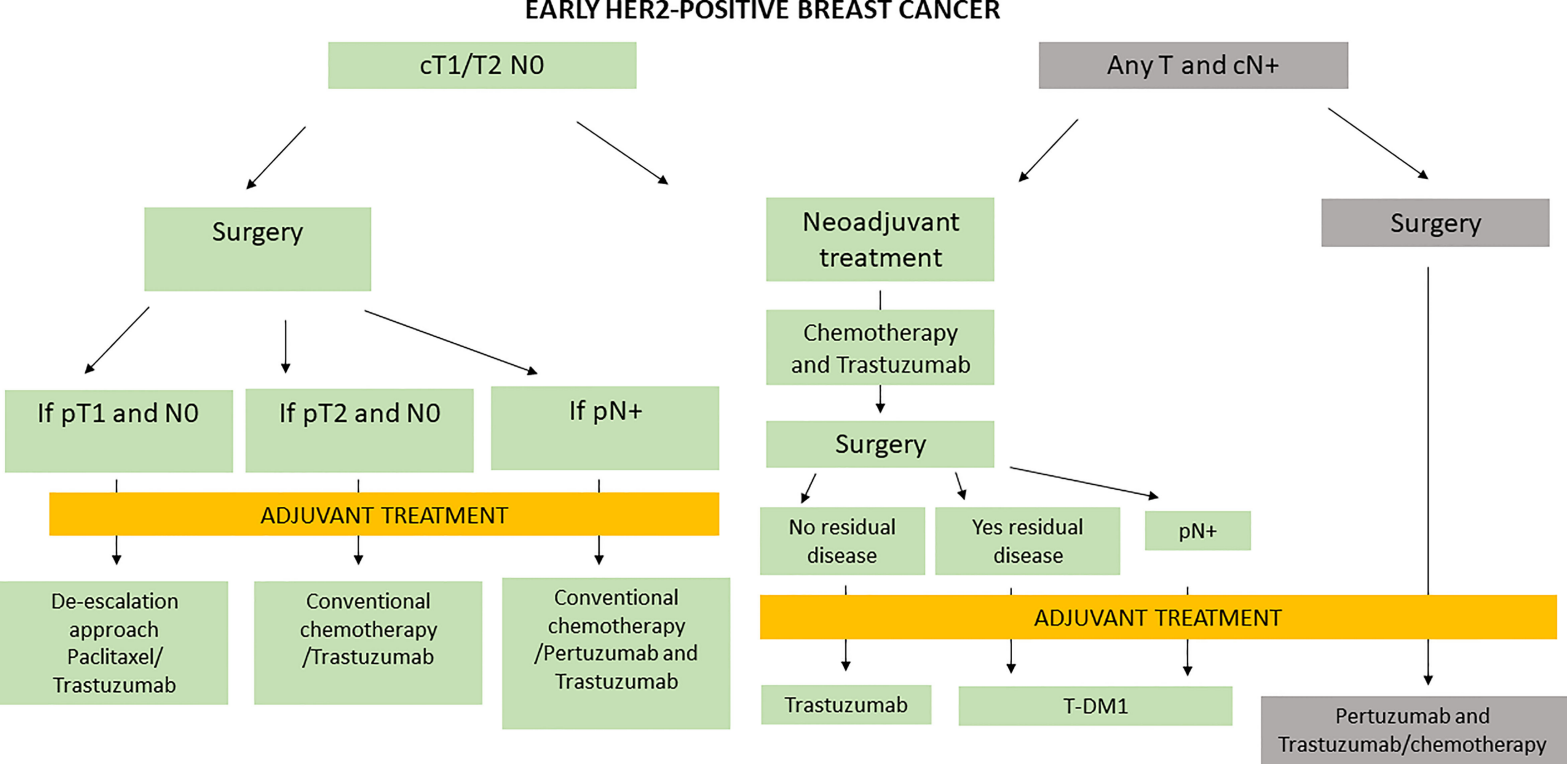
Management of early HER2-positive BC in Italy.

Nevertheless, in Italy, the management of early HER2+ BC is based both on results from clinical trials and on criteria of regulatory bodies, as presented in **Figure 1**.

Despite the improvements in HER2-positive early BC treatment (**Table 1**), many questions remain unanswered. Molecular heterogeneity within HER2-positive BC requires further investigation in order to develop valid biomarkers that may better define different risk subgroups of patients who might benefit from different treatment strategies at an early stage, translating to a better survival also in advanced disease. This approach is particularly valid after the introduction of other therapeutic arms in advanced disease such as trastuzumab deruxtecan or tucatinib, which are already under evaluation in earlier treatment settings.

**Table 1:** Summary of the principal clinical trials involving anti-HER2 agents for early-stage HER2-positive BC.

Anti-HER2 agents	Setting	Trial name	Patient characteristics	Adjuvant regimen	Primary endpoint	Results
Trastuzumab	Adjuvant	HERA	HER2-positive; node-positive disease (irrespective of pathological tumor size) or node-negative disease if tumor size >1 cm on pathological examination (*n* = 5102)	Chemo; 12 or 24 months T q3 vs. not	DFS	2 years of adjuvant T did not improve DFS compared with 1 year of T (HR 1.02, 95% CI 0.89–1.17)
Pertuzumab	Adjuvant	APHINITY	HER2-positive; node-positive or node-negative disease with tumor >1 cm (*n* = 4,805)	Chemotherapy + PH or Chemotherapy + Placebo/H	iDFS	91% 6-year IDFS for the pertuzumab group and 88% for the placebo group (HR 0.76, 95% CI, 0.64–0.91)
Pertuzumab + Trastuzumab	Neoadjuvant	NeoSphere	HER2-positive; operable (T2–3, N0–1, M0), locally advanced (T2–3, N2–3, M0 or T4a–c, any N, M0), or inflammatory (T4d, any N, M0) BC with primary tumors larger than 2 cm in diameter (*n* = 417)	H + docetaxel PH + docetaxel PH without chemotherapy P + docetaxel	pCR	29.0% (95% Cl 20.6–38.5) 45.8% (95% Cl 36.1–55.7) 16.8% (95% Cl 10.3–25.3) 24.0% (95% Cl 15.8–33.7)
	Neoadjuvant	WSG-TP-II	HER2-positive, HR+, cTlc-T4a-c (*n* = 207)	ET + PH Pac + PH	pCR	24% (95% Cl 16–34) 57% (95% Cl 47–67)
Lapatinib	Adjuvant	ALTTO	HER2-positive; node-positive disease or node-negative disease with pathologic tumor sizes 1 cm (*n* = 8,381)	1 year of adjuvant therapy with T, L, their sequence (T-H), or their combination (L + T)	DFS	16% reduction in the DFS hazard rate with L + T compared with T (HR, 0.84; 97.5% Cl, 0.7–1.02) and 4% reduction with T^L compared with T (HR, 0.96; 97.5% Cl, 0.80–1.15)
	Neoadjuvant	NeoALTTO	HER2-positive; tumor size greater than 2 cm (*n* = 455)	Lapatinib Trastuzumab Lapatinib + Trastuzumab	pCR	87% 93% 91%
	Neoadjuvant	NSABP B^l	HER2-positive, operable (*n* = 529)	AC ^ paclitaxel with L or T or L + T	pCR	T + L: 62% (95% Cl 54.3–68.8); L: 53.2% (95% Cl 45.4–60.3); T: 52.5% (95% Cl 44.9–59.5)
Neratinib	Adjuvant	ExteNET	HER2-positive; stage 1 to 3c who completed neoadjuvant or adjuvant chemotherapy plustrastuzumab (*n* = 2840)	Neratinib or placebo for 1 year	iDFS	5.1% 5-year iDFS benefit of neratinib versus placebo in HR+/1-year population (HR 0.73; 95% CI 0.57–0.92)
Trastuzumab emtansine	Adjuvant	KATHERINE	HER2-positive; residual invasive disease in breast or axilla at surgery after neoadjuvant therapy with taxane and trastuzumab (*n* = 1,486)	T-DM1 or trastuzumab for 14 cycles	iDFS	3-year iDSF 88.3% in the T-DM1 group and 77.0% in the trastuzumab group (HR 0.50; 95% CI 0.39–0.64)
	Neoadjuvant	KRISTINE	HER2-positive; stage II–III cT2–4 (>2 cm)/cN0–3/cM0 (>2 cm) (*n* = 444)	TDM-l/P x 6 Doc/Cb/PH x 6	pCR	44.4% 55.7% (95% Cl −20.5–2.0)

## Conclusions and Potential Clinical Implications

In summary, in Italy, trastuzumab is approved in neoadjuvant treatment for tumors >1 cm or node-positive BC patients and, in case of complete pathological response, in adjuvant therapy (NOAH trial); T-DM1 is approved and reimbursed as adjuvant therapy for residual disease after neoadjuvant treatment with trastuzumab (KATHERINE trial), whereas the double blockade with pertuzumab and trastuzumab is approved and reimbursed in Italy only in adjuvant setting for untreated high-risk (node-positive) HER2+ BC patients (APHINITY trial).

Nevertheless, the encouraging results from the NEOSPHERE trial, the limited number of patient recruitment, the absence of a blinded and centralized pathology analysis, and the chosen chemotherapy backbone prompted the Italian regulatory bodies to approve but not grant the reimbursability of pertuzumab plus trastuzumab in the neoadjuvant setting of therapy.

The European Commission approved the use of pertuzumab and trastuzumab in the neoadjuvant setting for HER2-positive BC, thus making the Perjeta regimen available to appropriate patients in the EU as early as possible. Neoadjuvant pertuzumab indication was approved in Europe in 2015 and, from an economic perspective, it received different recommendations from reference Health Technology Assessment (HTA) bodies. The National Institute for Health and Care Excellence (NICE) in the UK positively recommended the use of pertuzumab for NeoT, but received a negative recommendation from the National Centre for Pharmacoeconomics (NCPE) in Ireland. Innovative treatment access is critical to deliver high-quality healthcare, but sustainability must be considered, suggesting the importance of establishing a cost-effectiveness profile of pertuzumab in neoadjuvant therapy for early HER2-positive BC (45).

After the approval of a new therapeutic arm waiting for the reimbursement, the clinicians have to discuss with great care these treatments with patients, even if they always cannot offer them in public. Perhaps, the best strategy should be to tell all the possibilities in a truthful way. Oncologists should be able to handle difficult conversations like these in a way that balances principles of truth telling and transparency with a responsibility to avoid adding unnecessary distress to the patients.

Based on these lines of evidence, we have to discuss in Italy how we can ideally manage the early stage of HER2-positive BC according to approved and reimbursed drugs and the new drugs available in advanced disease. We can make decisions regarding initial therapy with HER2-targeting agents for early-stage disease based on the patient’s risk category and individual characteristics, along with considerations of potential therapy options in the setting of early-stage disease that also targets HER2 for women with later-stage disease. We use the most effective therapies for management of early-stage disease. We should not hold off on therapy in order to reserve it until patients develop metastatic BC. HER2-BC is highly curable because of the availability of these HER2-targeted therapies, so we treat patients fairly aggressively upfront to reduce the risk of them experiencing a stage IV recurrence. We consider factors such as hormone receptor co-expression, lymph node status, grade of the tumor, comorbidities, how healthy and functional an individual patient is, and whether the patient has pre-existing heart disease or any risk of heart disease. All of these factors are part of the decision regarding which regimen to recommend for a patient who is sitting before us with curable early-stage BC. Moreover, Italian oncologists to date do not have standard criteria to decide the best treatment to reserve to patients with higher-risk or node-positive HER+ BC. What are the criteria in selecting node-positive patients who will be treated with neoadjuvant treatment with trastuzumab or with adjuvant pertuzumab/trastuzumab? A survey involving the Italian oncology community is desired in order to conduct an analysis that explores the management of a single center of early node-positive HER2+ BC with the aim of having a report describing the likely discrepancy or uniformity in patient care. We do not forget that the Italian health system is inspired by the principles of universality, equality, and equity in access to care.

## Author Contributions

LS: first author and conceptualization. MP, CD’A, and TG: writing and figures. AT: supervision and final editing. All authors contributed to the article and approved the submitted version.

## Conflict of Interest

The authors declare that the research was conducted in the absence of any commercial or financial relationships that could be construed as a potential conflict of interest.

## Publisher’s Note

All claims expressed in this article are solely those of the authors and do not necessarily represent those of their affiliated organizations, or those of the publisher, the editors and the reviewers. Any product that may be evaluated in this article, or claim that may be made by its manufacturer, is not guaranteed or endorsed by the publisher.
